# pH-Responsive Carbon Foams with Switchable Wettability Made from Larch Sawdust for Oil Recovery

**DOI:** 10.3390/polym15030638

**Published:** 2023-01-26

**Authors:** Jia Tan, Jiaming Sun, Chunhui Ma, Sha Luo, Wei Li, Shouxin Liu

**Affiliations:** 1 College of Materials Science and Engineering, Northeast Forestry University, Harbin 150040, China; 2 Key Laboratory of Bio-Based Material Science and Technology of the Ministry of Education, Northeast Forestry University, Harbin 150040, China; 3 Engineering Research Center of Advanced Wooden Materials of the Ministry of Education, Northeast Forestry University, Harbin 150040, China

**Keywords:** switchable wettability, wood materials, carbon foams, oil recovery, oil cleanup

## Abstract

The global challenge of oil pollution calls for the efficient selective recovery of oil or organics from oil–water mixtures. A pH-responsive carbon foam (CF) made from liquefied larch sawdust (LLS) with switchable wettability was fabricated in this work. After grafted with poly 4-vinyl pyridine (P4vp), the CF obtained a switchable wettability surface, which allowed the CF to exhibit superhydrophilicity and superhydrophobicity at different pH levels, respectively. The results revealed that the pH-responsive CF possessed a three-dimensional (3D) spongy-like skeleton and porous structure with a diameter between 50 and 200 µm. Thus, the pH-responsive CF could absorb 15–35 g/g of oil/organics in a neutral aqueous solution at pH = 7 and desorb all the absorbate within 40 s after immersion in an aqueous solution at pH = 1. Moreover, only about 2.8% loss was observed for organic (chloroform) absorption and recovery after reusing up to 15 cycles, which indicated promising prospects in oil and organic recovery.

## 1. Introduction

With the rapid development of social industry and economy, the oily wastewater discharged from textile, petroleum, pharmaceutical industries, and human activities is increasing. This discharged wastewater contains a large number of organic compounds that are difficult to degrade naturally and have biological toxicity, which seriously threatens human health and ecosystem safety [1,2,3,4]. Therefore, developing practical solutions for collecting and recovering oil from water attracts worldwide attention. The hydrophilic oil–water separation absorbents are considered candidates for the field of oil–water treatment because it is relatively simple to prepare and easy to separate oil and organic solvents from oil–water mixtures. However, using non-renewable materials as feedstock makes them environmentally unfriendly [5,6,7]. Therefore, developing advanced absorbents based on renewable raw materials is a promising research direction [8].

Biomass-based polymeric materials are obtained through biological, chemical, and physical methods using renewable biomass, including crops, trees, or other plants, which have advantages traditional polymers do not have, such as being green, sustainable, and biodegradable [9,10,11,12]. Furthermore, biomass possesses abundant hydroxyl groups and phenolic hydroxyls, which is convenient to be selectively modified through many chemical reactions, including oxidation, esterification, etherification, sulfonation, and silylation, and thus is suitable to be applied in oil recovery [13,14]. For instance, Guan prepared a hydrophobic coating for hydrophilic balsa wood with a silanization agent via chemical vapor deposition (CVD) for separating oil and water. After chemical pretreatment, the wavy layered structure facilitated oil–water separation [15]. Except for using the bulk materials such as balsa wood mentioned above, employing liquefied wood to assemble varieties of products can meet different needs [16,17]. Nowadays, biodegradable sawdust waste has become one of the emerging materials in the field of oil–water separation [18]. Instead of using large pieces of economically valuable biomass feedstock, using inexpensive and readily available forestry waste that no longer has economic value has received more focused attention, such as corn straw fibers [19], natural leaf mesh [20], and cigarette filters [21]. Rajak synthesized porous activated carbon from the sawdust of the Indian white teak tree using an impregnation method. The activated carbon synthesized using phosphoric acid exhibited a higher adsorption capacity for the effective separation of petroleum from oil-in-water emulsions [22]. Zang obtained superhydrophobic/superoleophilic sawdust particles by mixing sawdust particles in a mixture of OTS-modified silica nanoparticles and polystyrene. The prepared superhydrophobic sawdust particles were placed in an oil–water mixture, and 3 mL of gasoline was collected after 90 min. Although the adsorption amount was quite high, the separation time was too long [23]. In our group, hydrophobic polymeric foam with a 3D interconnected porous honeycomb structure was prepared from larch sawdust waste and used for oil and organic separation [5]. However, its non-variable surface wettability made it unable to handle complex scenarios. For example, recovering oil is limited to compression, pumping, or heating. Additionally, its application is easily influenced by limitations of material and equipment. In addition, oils (especially high viscosity oil) easily adhere to the superhydrophobic surface, causing a reduction in absorption and difficult desorption [24]. It is urgent to find a way to deal with these challenges.

Recently, pH-responsive absorbents have provided new ideas for coping with this challenge. The superhydrophilicity and superhydrophobicity of the surface of pH-responsive CF polymers could be switched in response to external pH changes. The oil/organics in the oil–water mixture could be quickly absorbed by the pH-responsive absorbents when the external pH is neutral. The absorbate could be completely desorbed when the external pH changes to acidic or alkaline. With this approach, the adsorbent can easily be recovered without squeezing and burning; therefore, it is adapted to more complex and variable environments. For example, Jin fabricated a melamine sponge with pH-controlled wettability using a facile dip coating method. The coated sponge exhibited a rapid response involving reversibly switchable wettability from superhydrophilicity–superoleophobicity in acidic surroundings to superhydrophobicity–superoleophilicity under neutral or alkaline conditions [25]. Zhu prepared a pH-responsive graphene foam by modifying an amphiphilic copolymer that can rapidly absorb oil and organic solvents in neutral water at pH = 7 and precipitate the absorbed material at pH = 3. [26]. However, these porous sorbents with pH-responsive surfaces are usually obtained from non-renewable and expensive raw materials. The amount of research on adsorbents prepared from biomass is limited. Developing absorbents with switchable wettability for fast and efficient oil recovery from inexpensive and environmentally friendly feedstocks is required and makes sense [27].

Herein, a larch-sawdust-based pH-responsive CF with switchable wettability for fast and efficient oil absorption and recovery was prepared. It was fabricated via the carbonization of LLS-coated melamine foam and then grafted with poly 4-vinyl pyridine (P4vp). Poly 4-vinyl pyridine (P4vp) is a pH-responsive polymer and can be grafted on CF through interfacial-initiated atom transfer radical polymerization (ATRP). A large number of hydroxyl groups in CF provide reaction sites for the ATRP reactions of P4vp. The as-prepared pH-responsive CF could switch wettability between superoleophilicity and superoleophobicity in different pH mediums, providing the materials with excellent oil absorption and recovery ability. Specifically, this absorbent could efficiently absorb various oils, with high oil absorption capacity at neutral pH, and desorb the absorbed oil almost entirely in acidic water. Moreover, the pH-responsive CF reflected excellent recyclability in oil absorption and desorption, with almost no downward trend.

## 2. Materials and Methods

### 2.1. Materials

Larch sawdust (30–80 mesh, dried at 105 °C for 8 h) was provided by a local timber mill. (3-aminopropyl) trimethoxysilane (ATMS, 97%), 2-bromoisobutyryl bromide (2-Br, 98%), N,N,N′,N,′ N″-pentamethyldiethylenetriamine (PMDETA, 99%), and 4-vinyl pyridine (4-vp, 96%) were provided by Aladdin Industrial (Shanghai, China). Copper (I) bromide (99.9%) was provided by Aladdin Industrial (Shanghai, China) and purified with acetic acid, ethanol, and deionized water and then dried under vacuum before use. Dichloromethane (99.9%, water < 50 ppm), and pyridine (99.5%, water <50 ppm) were provided by Sinopharm Chemical Reagent (Peking, China). Melamine foam was purchased from the market, washed with ethanol and deionized water, and dried before use. Phenol (AR grade), methanol (AR grade), toluene (AR grade), acetone (AR grade), isopropanol (AR grade), and other chemicals were purchased from Kermel Chemical Corporation and used as received.

### 2.2. Characterization

A scanning electron microscope (SEM, Apreo S HiVac, Thermo Scientific, USA) operating at 5 kV was used to observe the morphology and element composition of the samples. X-ray photoelectron spectroscopy (XPS, K-Alpha Thermo Scientific, USA) measurements were performed with Al Ka radiation operating at 12 kV, full-spectrum scan pass energy of 150 eV in 1 eV steps, high-resolution-spectrum scan pass energy of 50 eV in 0.1 eV steps. A thermogravimetric analyzer (TGA, Q50 TA instruments, USA) was used to monitor the behavior of the samples with a heating rate of 10 °C/min under a nitrogen sphere. A drop-shape analyzer (DSA25, Krüss, Germany) was used to measure the wettability of the samples at room temperature using 10 µL droplet volumes.

### 2.3. Preparation of CF

Briefly, 40 g larch sawdust was added to a 120 mL phenol solution containing 2.0 mL of concentrated sulfuric acid and 3.4 mL of concentrated phosphoric acid. The reaction was stirred for 1 h after slowly heating up to 126 °C. Then, 100 mL of methanol was added to the reaction solution after it was brought to room temperature, and the liquefied larch sawdust solution (LLS) was obtained by conducting vacuum filtration of the mixture and washing the filter cake with 160 mL of methanol several times until the filtrate appeared colorless. Afterward, melamine foam samples (MS, 5 × 5 × 1 cm^3^, 0.1781 g) were immersed in the LLS for a few seconds and squeezed out the excess LLS under a pressure of 70 Nm. After standing overnight, the LLS-MS (1.6740 g) solutions were washed with ethanol and deionized water until neutral and then dried under vacuum. CF (0.6791 g) was obtained after carbonizing the LLS-MS under a nitrogen atmosphere at 700 °C for 30 min with a heating rate of 5 °C/min.

### 2.4. Preparation of pH-Responsive CF

The prepared CF was first placed in 300 mL of a toluene solution with 6.75 mL of ATMS and reacted at 120 °C for 6 h and then washed 3 times with toluene and dried at 120 °C for 1 h to remove the unreacted ATMS. Then, it was placed in 150 mL anhydrous dichloromethane containing 2% (*v*/*v*) of anhydrous pyridine in an ice-water bath, 2.70 mL of 2-Br was added dropwise, and the reaction was carried out at 0 °C for 1 h and then placed at room temperature for 12 h. The unreacted 2-Br was then washed with acetone and dried under vacuum. Finally, it was immersed in a 150 mL degassed solution of 1:1 (*v*/*v*) acetone and isopropanol containing 0.60 g copper (I) bromide and 3 mL PMDETA and reacted at 45 °C for 10 h after the addition of 15 mL 4-vinyl pyridine. The pH-responsive CF was obtained after washing with hydrochloric acid (pH = 2), deionized water, and ethanol three times to remove impurities and dried under vacuum.

### 2.5. Oil Absorption and Recovery Test

The oil desorption ability of CF-P4vp was evaluated using a cyclic oil absorbent (chloroform) in a neutral solution, followed by desorption in a pH = 1 aqueous solution. The CF-P4vp was immersed in chloroform and then taken out, the solution was weighed, and the reduced mass of chloroform was set as w_1_. Then, the CF-P4vp absorbed with chloroform was immersed in an aqueous solution with pH = 1, and the solution was left until chloroform was completely precipitated, and the mass of chloroform separated from the mixture was set as w_2_. After that, CF-P4vp was washed with water and dried under vacuum for the next turn of the desorption test. The oil desorption ratio (D) was calculated according to the following equation:

D = w_2_/w_1_ × 100% (1)

The oil absorption capacity of CF-P4vp was measured for various types of oil and organic solutions. The CF-P4vp was immersed in an oil or organic solution and then removed and weighed. The weight of CF-P4vp before oil absorption was set as m_0,_ and the weight of CF-P4vp after oil absorption was set as m_1_. The absorption capacity (Q) of CF-P4vp was calculated according to the following equation:

Q = (m_1_ − m_0_)/m_0_ (2)

## 3. Results and Discussion

### 3.1. Preparation and Characterization of pH-Responsive CF

**Figure 1:**
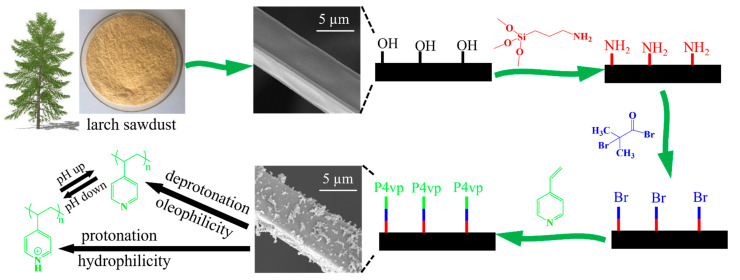
Schematic illustration of the synthesis of CF-P4vp.

The synthetic process of the pH-responsive CF by using larch sawdust as raw material is illustrated in Figure 1. The LLS was fabricated through the acid-catalyzed phenol-liquefaction and subsequently coated on the melamine sponge. The CF-containing hydroxyl groups were prepared after the carbonization of the coated foam at 700 °C for 30 min under a nitrogen atmosphere. The (3-aminopropyl) trimethoxysilane (ATMS) was grafted onto the surface of CF to form C-O-Si bonds via a dehydration condensation reaction between CF and the silanol hydroxyl group formed through the hydrolysis of ATMS with trace water in toluene. Subsequently, 2-bromobutyryl bromide was grafted to the end of -NH_2_ of ATMS via an acylation reaction in the process of atom transfer radical polymerization (ATRP) initiation. The pH-responsive polymer P4vp was polymerized onto the surface of CF after ATRP via interfacial initiation by using 4-vinyl pyridine as monomers. The CF-P4vp thus prepared could present switchable wettability through the reversible protonation and deprotonation of the pyridine groups of P4vp grafted on the surface. When the modified surface was immersed in a neutral oil–water mixture, the P4vp chains were deprotonated, resulting in a collapsed structure due to the absence of electrostatic repulsion between the pyridine groups. In this case, the P4vp chains were hydrophobic/oleophilic, and the surface of CF-P4vp presented as hydrophobicity/oleophilicity, causing the oil/organic solvent to be rapidly absorbed under capillary forces. In contrast, the deprotonated CF-P4vp became protonated upon immersion in acid water due to the combination of negatively charged pyridine groups with positively charged hydrogen ions. The P4vp molecular chain was stretched apart under the force of electrostatic repulsion of the protonated pyridine groups. In this case, the surface wettability of CF-P4vp exhibited hydrophilicity/hydrophobicity.

**Figure 2:**
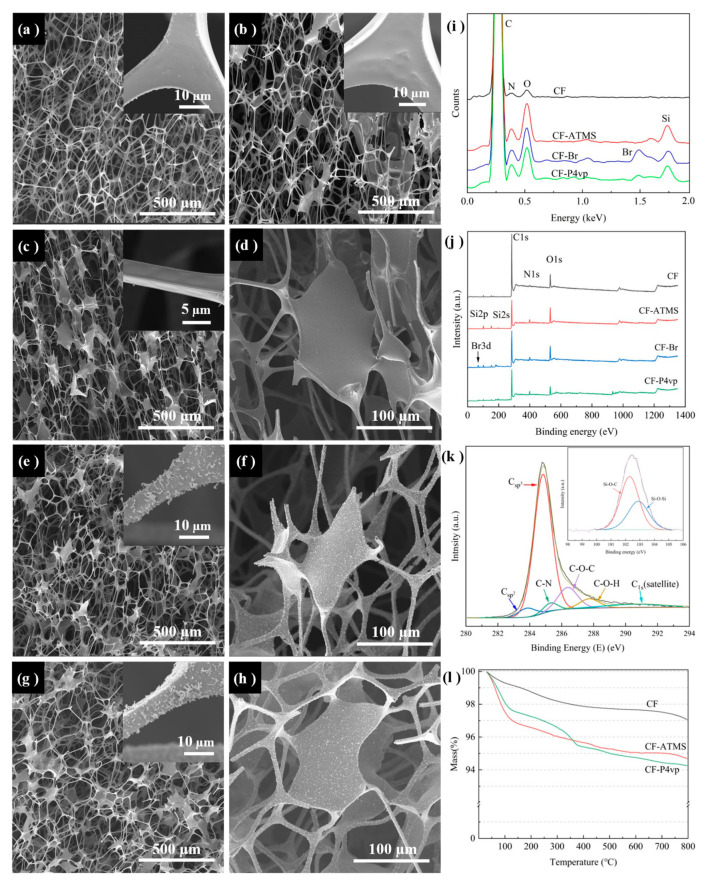
Characterization of the as-fabricated polymer foams: (**a**–**h**) SEM images of (**a**) MF, (**b**) LLS-MF, (**c**,**d**) CF, (**e**,**f**) CF-ATMS, and (**g**,**h**) CF-P4vp (pH-responsive CF); (**i**,**j**) EDS and XPS spectra of CF, CF-ATMS, CF-Br, and CF-P4vp; (**k**) XPS spectra of C^1s^ peak of CF and Si^2p^ peak of CF-ATMS (inset); (**l**) TGA curve of CF, CF-ATMS, and CF-P4vp under nitrogen.

Scanning electron microscopy (SEM) measurement was carried out to characterize the surface morphologies of the obtained CF before and after the treatments. As shown in Figure 2a, the melamine foam (MF) sample was a polymer with a smooth surface, a ligament diameter of about 10 µm, and a skeleton cell diameter between 100 and 500 µm. After coating with LLS, the surface of MF became significantly thicker and smoother. The width of the ligament increased from less than ten microns initially (Figure 2a) to more than ten microns (Figure 2b). CF was obtained after the carbonization of LLS-MF. The morphology of the CF remained unchanged, but its skeleton diameter shrank to about 50–200 µm, and the ligament decreased to about 5 µm (Figure 2c,d) [28]. A large amount of flocculent appeared on the surface of the CF-ATMS after grafting the (3-aminopropyl) trimethoxysilane (ATMS) (Figure 2e,f), C-O-Si bonds were formed through dehydration condensation reactions between CF and silanol hydroxyl groups, which were generated via the hydrolysis reactions of ATMS with trace amounts of water (0.03 wt%) in toluene (Figure 2k). The ATRP initiator 2-Br was grafted at the end of ATMS after an acylation reaction with -NH_2_. Finally, the P4vp was polymerized onto the surface following the ATRP reaction [29,30,31]. The successful grafting of P4vp on the framework surface resulted in a rougher surface of the pH-responsive CF, while the morphology remained unchanged (Figure 2g,h).

Energy-dispersive spectrometry (EDS) and X-ray photoelectron spectroscopy (XPS) were used to confirm the elemental change in the samples during the reactions. The EDS spectra of CF-ATMS and CF-Br showed the presence of Si and Br peaks, indicating that ATMS and 2-Br were successfully grafted to the surface. In contrast, the relative intensities of the corresponding Si and Br peaks on the spectra of P4vp were reduced, which is attributed to the grafting of P4vp (Figure 2i). The XPS spectrum of CF showed peaks of C (284.8 eV), N (398.6 eV), and O (532.1 eV) belonging to the CF skeleton (Figure 2j). The C^1s^ high-resolution spectrum of CF (Figure 2k) showed six peaks attributed to sp^3^ hybridized C-C (284.9 eV), sp^2^ hybridized C-C (283.9 eV), C-N (285.4 eV), C-O-C (286.4 eV), C^1s^ (satellite) (290.8 eV), and C-OH (287.8 eV) groups [32,33]. After the silylation modification of the CF surface using ATMS, the XPS spectra of CF-ATMS showed additional peaks of Si^2p^ (102 eV) and Si^2s^ (153 eV) (Figure 2j). The high-resolution spectrum of Si^2p^ on CF-ATMS can be divided into two peaks, 102.3 eV and 102.9 eV, which are attributed to the C-O-Si group generated by the dehydration of C-OH and Si-OH, and the O-Si-O group generated through the partial self-condensation reaction of silanol, respectively (Figure 2k; inset) [34]. The successful modification of initiator 2-Br was confirmed by the appearance of the Br3d peak in the XPS spectrum of CF-Br. Compared with C (79.17%) and Br (2.20%) contents of CF-Br, the content of C increased to 84.01%, and Br decreased to 1.04% of CF-P4vp, which demonstrated the successful modification of P4vp on CF-Br. The TGA curve of CF-Br showed a significant weight reduction between 180 and 360 °C, which was attributed to the pyrolysis of ATMS and 2-Br [23]. In the curve for CF-P4vp, an additional ~0.9 wt% mass loss occurred between 360 and 530 °C due to the pyrolysis acid-responsive group P4vp, indicating that about ~0.9 wt% P4vp grafted onto the pH-responsive CF.

### 3.2. Switchable Wettability of pH-Responsive CF

**Figure 3:**
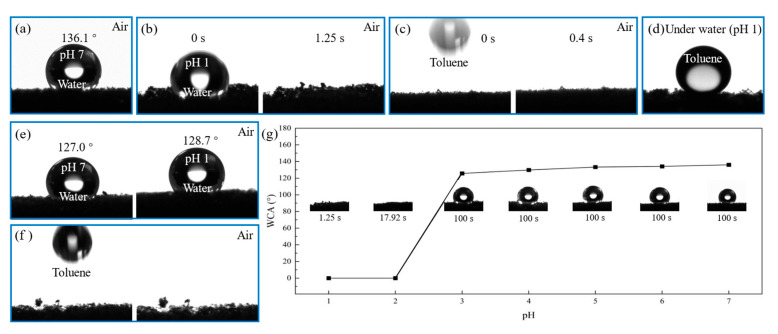
CA of CF and pH-responsive CF: (**a**) pH = 7 water drop stood on the surface of pH-responsive CF in the air; (**b**) pH = 1 water droplet was absorbed within 1250 ms in the air; (**c**) oil droplet was absorbed within 400 ms in the air; (**d**) oil droplet stood on the surface of pH-responsive CF under pH = 1 water; (**e**,**f**) unmodified CF; (**g**) comparison of resting time of water droplets with different pH values.

In the field of absorption, the surface of porous CF can absorb oil from oily water. CF possesses features of hydrophobic/oleophilicity and a high oil adsorption capacity of about 15–200 times its initial weight [24]. However, current CF-based adsorbents recover the adsorbed oil via compression or heat treatment, which is inefficient and may cause the destruction of the materials. The surface wettability of CF-P4vp at different pH aqueous solutions was investigated. The pH = 7 water droplet presented a stable, nearly spherical shape on the surface of pH-responsive CF with a water contact angle (WCA) of about 136.1° in the air (Figure 3a), which indicated that the pH-responsive CF had excellent hydrophobic properties in the neutral aqueous solution. In contrast, the pH = 1 water droplet was quickly absorbed by the pH-responsive CF within 1250 ms (Figure 3b), showing that the pH-responsive CF was superhydrophilic in the acidic aqueous solution. At the same time, the oil contact angle (OCA) was also tested. The pH-responsive CF showed superoleophilicity at a neutral pH. A toluene droplet was quickly and entirely absorbed by the pH-responsive CF within 400 ms (Figure 3c). Meanwhile, the OCA of toluene was about 146 ° underwater with pH = 1, indicating that the pH-responsive CF had superb oleophobicity in acidic solutions (Figure 3d). Comparing the pH-responsive CF with the unmodified CF (Figure 3e,f), it can be inferred that the modified pH-responsive CF possessed better hydrophobicity and obtained acid responsiveness. The variation in the WCA of the pH-responsive CF with different pH water droplets was investigated, as shown in Figure 3g. When the pH of the water droplets decreased from 7 to 3, the water droplets in the air remained spherical and unchanged even after one hundred seconds. However, as the pH continued to drop from 3 to 1, the water droplets were quickly absorbed. The time of water droplet absorption decreased from 17.92 to 1.25 s. This phenomenon showed that when pH was 3, the wettability of the pH-responsive CF rapidly changed.

The switchable wettability exhibited by the pH-responsive CF is attributed to the reversible protonation and deprotonation of the pyridine groups of P4vp grafted on the surface at different pH values [35]. When the pH-responsive CF was used in a neutral oil–water mixture, the pyridine group was deprotonated, and the P4vp molecular chain lost the electrostatic mutual repulsion inside to form a collapsed structure, thus exhibiting hydrophobic and oleophilic properties. Therefore, the pH-responsive CF exhibited superoleophilicity and good hydrophobicity, allowing organics to be rapidly absorbed through capillary action. In contrast, the deprotonated pyridine would be protonated after binding H^+^ in acidic water, and the P4vp molecular chains re-stretched under the electrostatic mutual repulsion, causing the pH-responsive CF to appear superhydrophilic and oleophobic. In summary, the grafted P4vp on the surface of CF allowed the pH-responsive CF to acquire the ability to exhibit switchable surface wettability at different pHs.

### 3.3. Application of pH-Responsive CF for Oil Recovery and Separation

**Figure 4:**
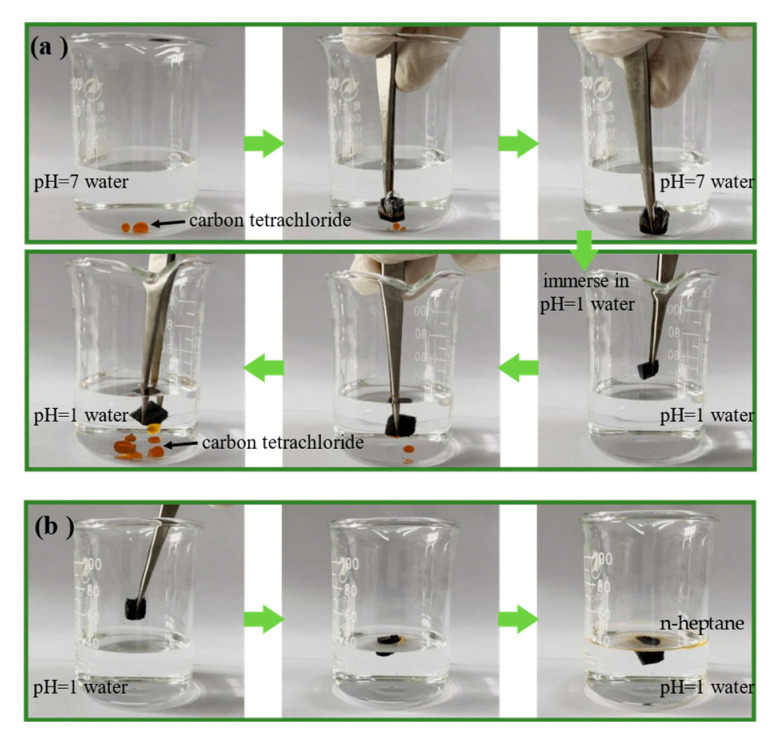
(**a**) Oil (carbon tetrachloride dyed with Sudan III) absorption in pH = 7 water and desorption in acidic water (pH = 1) by pH-responsive CF; (**b**) oil (n-heptane dyed with Sudan III) desorption by pH-responsive CF in acidic water (pH = 1).

The above pH-responsive wettability of the pH-responsive CF indicates that they could be applied to oil adsorption and desorption. First, the pH-responsive CF was immersed in an oil–water mixture with carbon tetrachloride (dyed with Sudan III) at pH = 7. When the pH-responsive CF was in contact with carbon tetrachloride located underwater, carbon tetrachloride was rapidly absorbed because the pH-responsive CF was hydrophobic and superoleophilic at pH = 7. Subsequently, the CF absorbed with carbon tetrachloride was removed from the neutral aqueous solution and immersed into the acidic aqueous solution at pH = 1. Due to the gradual protonation of the pyridine group on P4vp, the pH-responsive CF exhibited superhydrophilicity and oleophobicity, water gradually entered the interior of the CF, and carbon tetrachloride was rapidly desorbed (Figure 4a). Similarly, the pH-responsive CF absorbed with n-heptane (dyed with Sudan III) was added to an acidic aqueous solution at pH = 1. The n-heptane was desorbed and formed an oil film on the aqueous solution because the density of n-heptane is lower than water (Figure 4b). The above performance confirms the intelligent oil absorption and recovery capability of the pH-responsive CF in the two solutions with pH of 1 and 7, respectively.

**Figure 5:**
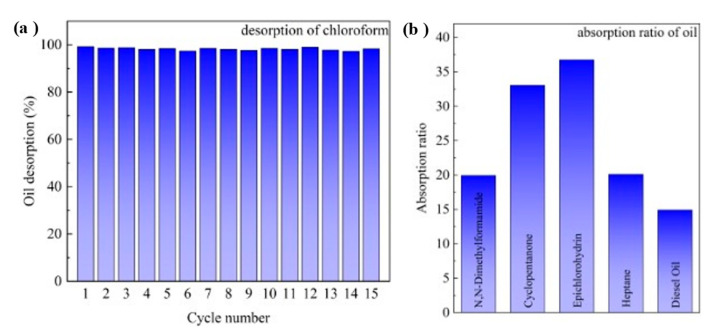
(**a**) Desorption recyclability of pH-responsive CF in pH = 1 water; (**b**) absorption capacity of various oils of pH-responsive CF.

The pH-responsive CF takes advantage of the switchable wettability and thus presents excellent recyclability upon absorbing and desorbing oils, which is highly demanded in practical applications. The recovery performance of the pH-responsive CF was verified by repeatedly performing oil absorption under neutral conditions and oil desorption under acidic conditions using chloroform as absorbate. The chloroform was absorbed by the CF, desorbed in an acidic solution, washed with deionized water to remove the acidic water, and dried under vacuum. After performing this operation 15 times, there was only about 2.8% loss of oil desorption, as shown in Figure 5a. The absorption ability of the pH-responsive CF for various oils and organics was investigated, and the results are shown in Figure 5b. Some oil and organic solvents containing common functional groups such as N,N-dimethylformamide, cyclopentanone, epichlorohydrin, heptane, and diesel oil were tested as model adsorbates. To study the absorption capacity quantitatively, the absorption ratio was defined as the weight of an absorbed substance per unit of weight of the dried pH-responsive CF. It was found that the pH-responsive CF exhibited good absorption capacity for these oils, with a typical absorption capacity of 15–37 times its weight. Compared with various switchable wettable surfaces published in the past (Table 1), the P4vp polymer revealed excellent separation efficiency and acceptable preparation costs. The preparation process and the final polymer are also simpler than other response types. Therefore, P4vp grafting layers could give polymeric absorbents a competitive advantage in the field of oil–water separation.

## 4. Conclusions

In summary, a larch-sawdust-based pH-responsive carbon foam was fabricated by grafting a P4vp polymer on the surface of the CF. The obtained pH-responsive CF possessed a spongy-like 3D skeleton structure and showed excellent reversible and switchable wettability at different pH values. Due to a large number of hydroxyl groups in CF, P4vp was easily and firmly grafted onto the CF. Thus, such an intelligent surface of P4vp caused the pH-responsive CF to quickly absorb the oil (carbon tetrachloride and n-heptane) in a neutral (pH = 7) solution and ultimately release it in an acidic (pH = 1) solution. Consequently, the pH-responsive CF revealed a high absorption capacity (15–37 g/g) and a quick oil recovery ability (40 s). Only about 2.8% loss was observed for organic (chloroform) absorption after reusing up to 15 cycles in the recycling tests. This pH-responsive CF is a promising absorbent with the potential for fast and efficient oil recovery.

## Figures and Tables

**Table 1:** Comparison of various polymeric absorbents.

Response Type	Polymer	Polymerization	Method	WCA (°)	Separation Efficiency (%)	Absorption Capacity (g/g)	Cost	Ref.
Hygro	Waste potato residue powder	Natural waste	Spray-coating	0	>96.5	-	low	[36]
Magnetic and Thermo	PNIPAM	SI-ATRP	Surface grafting	-	>80	-	medium	[37]
Gas	PDEAEMA	SI-ATRP	Surface grafting	113	>92	-	high	[38]
Light	Crosslinked azobenzene-containing polymer	Suspension-free radical polymerization within situ crosslinking	In situ crosslinking	-	-	~15	high	[39]
pH	PDMA-co-PTMSPMA-co-PDMAEMA	Free radical polymerization	Dip-coating	150	>99	25~26	high	[40]
pH	P4vp	Polymer	ATRP	155	~97	60–120	medium	[30]
pH	Larch sawdust	Natural waste	Liquefaction and ATRP	136	~97.2	15–35	medium	This work

## Data Availability

The data presented in this study are available on request from the corresponding author.
